# QTc prolongation risk among patients receiving oral targeted antineoplastic medications: A real-world community-based oncology analysis

**DOI:** 10.3389/fonc.2023.1098333

**Published:** 2023-03-10

**Authors:** David J. Reeves, Molly Russell, Vijay U. Rao

**Affiliations:** ^1^ Department of Pharmacy Practice, College of Pharmacy and Health Sciences Butler University, Indianapolis, IN, United States; ^2^ Franciscan Physician Network, Franciscan Health, Indianapolis, IN, United States; ^3^ Department of Pharmacy, Atrium Health Carolinas Medical Center, Charlotte, NC, United States; ^4^ International CardioOncology Society Center of Excellence, Indiana Heart Physicians, Indianapolis, IN, United States

**Keywords:** QTc prolongation, cardio-oncology, tyrosine kinase inhibitor, real-world analysis, risk factors

## Abstract

**Introduction:**

Thirty oral targeted antineoplastic agents are associated with prolongation of the QT interval. However, limited data exists regarding QTc prolongation and associated risk factors in the ambulatory oncology setting.

**Methods:**

This retrospective study was completed to describe QTc prolongation incidence among patients receiving oral targeted tyrosine kinase inhibitors (TKI) and identify potential risk factors in the ambulatory community-based oncology clinic.

**Results:**

Of the 341 patients identified as receiving oral TKI, 49 with a baseline and follow-up ECG were included. The incidence of QTc prolongation (QTc > 470 ms in males, QTc > 480 ms in females, or >20 ms increase in QTc from baseline) was 24%. Three patients developed significant QTc prolongation (QTc >500 ms or >60 ms increase in QTc from baseline). No patients discontinued therapy primarily due to QTc prolongation or experienced symptomatic torsades de pointes. Analysis of risk factors demonstrated that patients with QTc prolongation were more likely to receive concomitant therapy with a loop diuretic (41% vs 11%, respectively, p=0.029).

**Discussion:**

The frequency of QTc prolongation may be higher in the real-world setting than that observed in clinical trials; however, continuation of therapy may be possible. Patients receiving concomitant loop diuretics should be monitored more closely for QTc prolongation and electrolyte abnormalities.

## Introduction

Prolongation of the QT interval, a representation of ventricular repolarization, has long been associated with a potential increase in the risk of ventricular arrhythmia, specifically torsades de pointes (TdP). Over 250 drugs in clinical use have the potential to prolong the QT interval, 30 of which are oral antineoplastic targeted agents (1) that are being incorporated into the therapy of multiple malignancies. Retrospective analyses of these agents, predominantly tyrosine kinase inhibitors (TKIs), have demonstrated an incidence of QT corrected for heart rate (QTc) prolongation of 5.8% to 28%, depending on the definition of QTc prolongation used (2, 3). Significant QTc prolongation (QTc greater than 500 ms) was observed in 1.4% (2). The proposed mechanisms for TKI-induced QT prolongation include inhibition of the rapid component of the delayed rectifier potassium channel (IK_r_) or phosphoinositide 3 kinase (PI3K) signaling pathway (4). Currently, there is a lack of data regarding the overall incidence of QTc prolongation specifically among those antineoplastic agents most closely associated with increases in the QT interval.

Utilizing known risk factors, risk stratification scores have been developed; however, these risk scores are specific to the inpatient, acute care setting (5–7). Ambulatory cancer patients are unique in that they are usually medically stable while having a potentially exaggerated risk above and beyond ambulatory patients without cancer. In this population, additional risks such as more frequent electrolyte abnormalities or dehydration due to anticancer therapy induced diarrhea and/or nausea/vomiting may be present. In an effort to describe TKI induced QTc prolongation in a real-world setting, we analyzed QTc intervals of patients treated with oral TKIs known to prolong the QTc interval at a community-based oncology clinic.

## Methods

This was a retrospective analysis of adult patients over age 18 years initiated on an oral TKI known to prolong QTc between January 2017 and March 2021 at a large, community-based hospital, Franciscan Health, Indianapolis, Indiana. TKIs defined as having a known risk or possible risk for TdP by the Arizona Center for Education and Research on Therapeutics and available through their website (crediblemeds.org) were included in the analysis (**Table 1**). Known risk is defined as drugs that both prolong QT interval and are clearly associated with a known risk of TdP, while possible risk is defined as drugs that cause QT prolongation but lack evidence regarding a risk for TdP (1). Patients were excluded if they had a history of congenital long QT syndrome or atrial fibrillation, had an ICD or pacemaker, or did not have a baseline and follow-up electrocardiogram (ECG) documented and able to be queried in the electronic health record during therapy. The study was approved by the local Institutional Review Board.

**Table 1:** Tyrosine kinase inhibitors classified as being associated with QTc prolongation by crediblemeds.org.

Drug	Risk	Drug	Risk
Bosutinib	PR	Lenvatinib	PR
Cabozantinib	PR	Midostaurin	PR
Certinib	PR	Mobocertinib	KR
Cobimetinib	PR	Nilotinib	PR
Crizotinib	PR	Osimertinib	PR
Dabrafenib	PR	Pazopanib	PR
Dasatinib	PR	Ribociclib	PR
Encorafenib	PR	Selpercatinib	PR
Entrectinib	PR	Sorafenib	PR
Gilteritinib	PR	Sunitinib	PR
Imatinib	PR	Vandetanib	KR
Ivosidenib	PR	Vemurafenib	PR
Lapatinib	PR		

KR, Known risk (defined as drugs that both prolong QT interval and are clearly associated with a known risk of TdP); PR, Probable risk (defined as drugs that cause QT prolongation but lack evidence regarding a risk for TdP).

Patients were reviewed from therapy initiation until discontinuation or for a total of six months if the drug was not discontinued before September 2021. The primary endpoint of the study was the incidence of QTc prolongation. Key secondary endpoints included the incidence of significant QTc prolongation, QTc prolongation resulting in therapy discontinuation, and the identification of potential risk factors for QTc prolongation. QTc prolongation was defined as a QTc greater than 470 ms in males, greater than 480 ms in females or greater than 20 ms increase in QTc from baseline. Significant QTc prolongation was defined as a QTc above 500 ms or a greater than 60 ms increase in QTc from baseline. QTc values at baseline and first follow-up were collected. QTc measurements were ECG machine derived and based upon the Bazett formula as reported in the electronic health record. Risk factors collected from the medical record were age, sex, use of loop diuretics, glomerular filtration rate, past medical history documented in the patient problem list (hypertension, diabetes, coronary artery disease, heart failure, myocardial infarction), and use of other drugs known to prolong QTc intervals.

The primary outcome was analyzed using descriptive statistics. Secondary outcomes were analyzed using the Mann-Whitney U test for continuous, non-parametric data, student t-test for continuous, parametric data, and the chi-squared test or Fisher’s exact test, as appropriate for nominal data. P-values of < 0.05 were considered statistically significant. All statistical analyses were performed with IBM SPSS Statistics 25 (IBM Corp, Armonk, Ny) software.

## Results

Three hundred and forty-one patients receiving TKIs with a risk for QTc prolongation were identified and 49 patients met inclusion criteria. Reasons for exclusion were lack of baseline and follow-up ECG documented and able to be queried within the health record (n=215), therapy never initiated (n=20), history of atrial fibrillation (n=7), duplicate entry (n=4). In total, included patients received one of thirteen different TKIs (**Table 2**), the most common being dasatinib (n=9, 18%), pazopanib (n=7, 14%), and sunitinib (n=6, 12%). Fifty-five percent of the population were female and a majority had a cardiac comorbidity (**Table 2**). Four patients had a prolonged QTc interval at baseline. The median number of days between the baseline ECG and follow-up ECG was 38 days.

**Table 2:** Baseline Characteristics.

Baseline Characteristic	N=49
Age (years), mean ± SD	64 ± 12
Female – n (%)	27 (55)
GFR ≤ 50​, n (%)	7 (14)
Concomitant loop diuretic – n (%)	9 (18)
Hypertension – n (%)	32 (65)
Diabetes – n (%)	14 (29)
Coronary artery disease – n (%)	10 (20)
Heart failure – n (%)	7 (14)
History of myocardial infarction – n (%)	2 (4)
QT Interval (ms)– mean ± SD	395 ± 43
QTc Interval (ms) – mean ± SD	436 ± 29
QTc prolongation at baseline – n (%)	4 (8)
Tyrosine kinase inhibitor medication – n (%)	
Bosutinib	2 (4)
Dabrafenib	1 (2)
Dasatinib	9 (18)
Encorafenib	4 (8)
Lenvatinib	4 (8)
Midostaurin	1 (2)
Nilotinib	4 (8)
Osimertinib	6 (12)
Pazopanib	7 (14)
Ribociclib	2 (4)
Sorafenib	2 (4)
Sunitinib	6 (12)
Vemurafenib	1 (2)

GFR, glomerular filtration rate; n, number, SD, standard deviation.

The primary endpoint, incidence of QTc prolongation (QTc > 470 ms in males, QTc > 480 ms in females, or >20 ms increase in QTc from baseline) occurred in twelve patients (24%). TKIs received by patients experiencing QTc prolongation can be found in **Table 3**. Of these, four patients (8%) had a QTc greater than the defined threshold (470 ms in males or 480 ms in females) and eleven (22%) had a greater than 20 ms increase in QTc from baseline (three patients experienced both a QTc above the threshold and greater than 20 ms increase from baseline). Significant QTc prolongation (QTc above 500 ms or greater than 60 ms increase in QTc from baseline) occurred in three patients (6%) of which one patient had a QTc greater than 500 ms and two patients had both a QTc above 500 ms and a greater than 60 ms increase from baseline. See **Table 4** for details of individual patients experiencing significant QTc prolongation. Among those with significant QTc prolongation, two were receiving sunitinib and the third received sorafenib. One patient was receiving concomitant azithromycin. All patients had the TKI held upon discovery of the significantly prolonged QTc interval. At the time of significant QTc prolongation, the patient who was also receiving azithromycin was hypokalemic (2.6 meq/L) and had therapy resumed once normokalemia was restored and azithromycin therapy was completed. One patient experienced dehydration, acute kidney injury, and elevated transaminases at the time of significant QTc prolongation and had therapy resumed when the acute issues resolved. The last patient with significant QTc prolongation developed acute kidney injury and acute hepatic failure and the therapy was discontinued due to poor overall tolerability. No patients discontinued therapy primarily due to QTc prolongation or experienced symptomatic TdP.

**Table 3:** Kinase inhibitor medications utilized in patients experiencing QTc prolongation.

Drug	Number of Patients
Dabrafenib	1
Dasatinib	3
Encorafenib	1
Lenvatinib	2
Nilotinib	1
Pazopanib	1
Sunitinib	2

**Table 4:** Patient details for those developing significant QTc prolongation.

	78 year old male	59 year old female	75 year old male
**Tyrosine kinase inhibitor**	Sunitinib	Sunitinib	Sorafenib
**Baseline QTc**	468 ms	449 ms	453 ms
**Follow up QTc**	519 ms	521 ms	527 ms
**Receiving Loop Diuretic**	Yes – Torsemide 10 mg daily	No	Yes – Furosemide 60 mg daily
**Past Medical History/other risk factors**	Hypertension, diabetes, coronary artery disease Developed acute kidney injury and acute hepatic failure	Hypokalemia (K 2.6 meq/L)	Diabetes Dehydration, acute kidney injury, and elevated transaminases
**Concomitant QTc Prolonging Drugs**		Receiving course of azithromycin	
**Outcome**	Therapy held and not restarted due to poor overall tolerability	Therapy held and resumed once normokalemia was restored and azithromycin therapy was completed	Therapy held and resumed when acute issues resolved

Analysis of potential risk factors between those developing prolonged QTc and those without prolonged QTc intervals demonstrated that the two groups were similar overall, including in baseline demographics, relevant past medical history, baseline QTc interval, and potassium levels at baseline and first follow-up (**Table 5**). One significant difference observed between groups was the more frequent use of loop diuretics in the prolonged QTc interval group (42% *vs*. 11%, respectively, p=0.029). In the group without QTc prolongation all patients receiving loop diuretics also had a history of heart failure while among those with QTc prolongation, 2 of the 5 patients receiving a loop diuretic also had a history of heart failure. Loop diuretics received by those with QTc prolongation included torsemide in 3 patients (doses ranged from 10 mg to 40 mg daily) and furosemide in 2 patients (dosed at 40 mg and 60 mg daily). Among those without QTc prolongation, loop diuretics received included furosemide in all 4 patients (doses varied from 20 mg to 80 mg daily). Four patients (11%) without QTc prolongation were receiving one concomitant QTc prolonging medication at baseline compared to 1 patient (8%) who experienced QTc prolongation. Among the three patients experiencing significant QTc prolongation, two were receiving a loop diuretic, one had a history of hypertension, two had a history of diabetes, and one had a history of coronary artery disease.

**Table 5:** Analysis of risk factors for QTc prolongation.

Risk Factor Analysis	Non-Prolonged QTc N=37 n (%)	Prolonged QTc N=12 n (%)	p-value
Age, years, mean ± SD	64 ± 13	65 ± 10	0.760
Female, n (%)	20 (54)	7 (58)	0.796
Concomitant loop diuretic, n (%)	4 (11)	5 (42)	0.029
Number of concomitant QT prolonging medications at baseline, median (IQR)	0 (0)*	0 (0)^	0.807
GFR ≤ 50 ml/min^¥^, n (%)	6 (16)	1 (8)	0.665
Hypertension, n (%)	24 (65)	8 (67)	0.233
Diabetes, n (%)	10 (27)	4 (33)	0.721
Coronary artery disease, n (%)	6 (16)	4 (33)	0.233
Heart failure, n (%)	5 (14)	2 (17)	1.0
History of myocardial infarction, n (%)	1 (3)	1 (8)	0.434
Baseline QTc (ms), mean ± SD	437 ± 31	432 ± 27	0.614
Baseline QTc prolongation, n (%)	4 (11)	0 (0)	0.560
Baseline potassium, meq/L, median (IQR)	4.0 (0.6)	4.1 (0.4)	0.100
Potassium at time of follow-up ECG	4.0 (0.6)	4.2 (1.1)	0.625

GFR, glomerular filtration rate; IQR, Interquartile range; n, number; SD, standard deviation.

*4 patients were receiving one concomitant QT prolonging medication at baseline; the remainder were not receiving any concomitant QT prolonging medications.

^1 patient was receiving one concomitant QT prolonging medication at baseline; the remainder were not receiving any concomitant QT prolonging medications.

^¥^GFR cut off utilized by Berger, et al. (7) was 50 ml/min, while Tisdale et al. (6) utilized a cut off of 30 ml/min. This analysis utilized the more conservative estimate of 50 ml/min.

## Discussion

Despite almost a quarter of patients developing QTc prolongation while receiving TKIs with a known or possible risk for TdP, only 6% experienced significant QTc prolongation. The common terminology criteria for adverse effects (CTCAE) are often utilized to characterize adverse effects in oncology clinical trials and defines QTc prolongation as grade 1 if QTc 450-480 ms, grade 2 if QTc 481-500 ms, grade 3 if QTc greater than 500 ms or greater than 60 ms change from baseline, and grade 4 if a patient experiences TdP, polymorphic ventricular tachycardia, or signs/symptoms of a serious arrhythmia (8). In a retrospective study of 363 patients from four centers in the Netherlands and Italy who were receiving any TKI for solid tumors, there was a significant increase in CTCAE grade after starting a TKI, with 33 demonstrating an increase in grade (p=0.0003) (2). The majority (31) increased from grade 0 (less than 450 ms) to grade 1 or higher while only 2 increased from grade 1 to grade 2 or 3. The authors defined high-risk QTc as an interval above 470 ms, which was observed in 1.7% at baseline and 5.8% while on therapy and twenty patients transitioned from low risk to high risk. Only 1.4% developed a QTc greater than 500 ms. The median time to follow up ECG was 43 days. Variables identified that increase risk for progression to a higher CTCAE grade included age and hypokalemia. Variables associated with transitioning to the high-risk category included age and concomitant use of other QTc prolonging medications. Our study had more than twice the risk for developing a QTc interval greater than 500 ms and a slightly higher rate of QTc above 470 ms (5.8% *vs* 8% in our analysis). This may be due to the fact that we included only those TKI with the highest risk while the described study included any TKI, including some without a QTc prolongation risk such as erlotinib and gefitinib; however, the majority of the patients received those with a QTc prolongation risk (imatinib, lapatinib, pazopanib, sunitinib, sorafenib, and vemurafenib).

A more recent retrospective analysis of QTc prolongation risk among patients treated with TKI also looked at the incidence of QTc prolongation with all TKI, regardless of QTc prolongation risk (3). This analysis included 618 patients with 902 TKI administrations and defined QTc prolongation as a QTc interval of 450 ms or higher in men or 470 ms or higher in women. In total 29% experienced QTc prolongation, most commonly with nilotinib (39%) and dasatinib (41%). Risk factors for QTc prolongation included age, obesity, and history of hypertension, diabetes, hyperlipidemia, or congestive heart failure. QTc prolongation to an interval of 500 ms or greater or a 60 ms or higher increase from baseline occurred in 5% and 7% of TKI administrations, respectively. Increase in QTc interval from baseline were statistically significant with dasatinib, imatinib, nilotinib, sunitinib, and pazopanib. Sixty-eight percent of patients with QTc prolongation had their therapy discontinued in response (21% were switched to a different TKI, 46% were switched to a different type of treatment such as chemotherapy, radiotherapy, or bone marrow transplant), 13.5% had doses reduced, and 8.1% had therapy temporarily withheld. Ventricular tachycarida, TdP, or sudden cardiac death occurred in 5.4% of those experiencing QTc prolongation. Though definitions utilized in this study were different from those utilized here, the incidences of QTc prolongation were similar to our study. Of note, concomitant QTc prolonging medication use was not analyzed in this study. Our clinic’s standard of care is for every patient to undergo pharmacist review of their medication profile to minimize concomitant QTc prolonging medications, resulting in the majority (90%) in our study without any concomitant QTc prolonging medications.

In a meta-analysis of phase 2 and 3 trials with vascular endothelial growth factor receptor targeted TKIs, the relative risk for all CTCAE grades of QTc prolongation (QTc greater than 450 ms) was found to be 8.66 [95% confidence interval (CI): 4.92-15.2, p < 0.001] among the 6,548 patients from 18 trials (9). The majority of included trials (15 of 18 included trials) required QTc monitoring at baseline and periodically throughout the study (2 trials did not include QTc monitoring in the protocol and QTc monitoring practices was not addressed in 1 trial). The relative risk for high CTCAE grade QTc prolongation (QTc above 500 or serious arrhythmias) was 2.69 (95% CI: 1.33-5.44, p=0.006). All grade QTc prolongation was observed in 4.4% of the population, while high grade was observed in 0.83%. Agents included in this analysis were sunitinib, sorafenib, pazopanib, vandetanib, cabozanib, ponatninb, and regorafenib. When individual agents were analyzed, vandetanib and sunitinib demonstrated a significant increase in all grade QTc prolongation and pazopanib and axitinib use resulted in no increase in QTc prolongation. Interestingly, the relative risk for all grade QTc prolongation increased as the vandetanib dose increased (relative risk 10.6 at 300 mg *vs*. 4.83 at 100 mg). Trials with a longer treatment duration did not result in a higher risk of QTc prolongation. Despite a stricter definition of QTc prolongation, the rate of QTc prolongation in our study was higher than in this meta-analysis (24% *vs*. 4.4%, respectively) as was high grade QTc prolongation (1.4% *vs*. 0.83%). In our analysis, a total of 19 patients received vascular endothelial growth factor directed TKI, of which six (31.5%) developed QTc prolongation. These differences demonstrate the potential disparities witnessed between results obtained in controlled clinical trials and those observed in real-world clinic settings.

Real-world analyses, like this, are largely complementary to the data from randomized clinical trials in that they often include patients who may have been excluded from the clinical trials and are representative of the population treated in oncology clinics. As such, the increased rate of QTc prolongation in this analysis may be more typical of what is happening in clinic where patients likely have more risk factors, increased comorbidities, and decreased control over factors such as concomitant use of additional QTc prolonging medications. Likewise, real world analyses identify opportunities for improvement in practice such as the lack of follow-up ECG in almost 2/3 of the patients identified for inclusion in this analysis and the delay in time to follow up ECG (38 days in this study and 43 days in the previously discussed retrospective study). According to a recent state-of-the-art review, a follow-up ECG should be considered 14 days after staring therapy in all patients receiving TKI with an increased risk for QTc prolongation (10).

Due to the risk for QTc prolongation associated with many oral TKI, multiple recommendations exist for the management of patients receiving these therapies (11–13). The International Cardio-Oncology Society consensus statement for defining cardiovascular toxicities of cancer therapies recommended action be taken if the QTc interval increased above 500 ms as the risk of TdP is low below this threshold, even if QTc increases more than 60 ms from baseline but QTc remains below 500 ms (11). The recommendations included a consideration for change in therapy when QTc exceeds 500 ms and to reassess QTc as changes in clinical status occur. Recently published European Society of Cardiology (ESC) guidelines on cardio-oncology, developed in association with the European Hematology Association, the European Society for Therapeutic Radiology and Oncology, and the International Cardio-Oncology Society provided comprehensive recommendations for the management of QTc prolongation associated with antineoplastic therapies (12). Evaluation of the ECG is recommended at baseline and once steady-state drug levels have been achieved in patients starting therapy known to cause QTc prolongation and routine ECG monitoring is recommended in patients at risk for QTc prolongation after dose increases, if electrolyte imbalances occur, or if concomitant QTc prolonging medications are added. Risk factors for QTc prolongation identified in these guidelines include concomitant QTc prolonging medications, bradyarrhythmia, electrolyte imbalance, renal or hepatic dysfunction with resultant inadequate antineoplastic dose adjustments, acute myocardial infarction, age greater than 65, prolonged baseline QTc, family history of sudden death, female sex, personal history of syncope or drug induced TdP, and pre-existing coronary artery disease, heart failure, or left ventricular hypertrophy.

Much of the data regarding patient specific risk factors are from the acute care setting where validated risk scores have been developed and implemented in practice (5–7). Though it is important to identify and address modifiable risk factors prior to starting drugs known to prolong QTc interval, the utility of such risk factors in determining the risk of a patient in the ambulatory setting may be limited as it is unclear if those factors identified in the acute care setting translate to the ambulatory setting (13). Likewise, routine QTc monitoring may not be prognostic in all patients as most with prolonged QTc intervals never experience TdP and many developing TdP have normal QT intervals prior to the event (4, 14). Unlike therapies for other diseases, many of the TKI for the treatment of malignancy lack alternatives and strict guidelines for QTc prolongation management may restrict the use of potentially life-prolonging medications (4). Our analysis of risk factors demonstrated that more patients developing QTc prolongation received concomitant loop diuretics. Two of the three risk scores developed for the acute care setting included loop diuretics in their scoring system (6, 7); however, this potential risk has not been demonstrated, to our knowledge, in the community-based outpatient setting among patients receiving TKI with a risk for QTc prolongation. Our data suggest that patients requiring use of a loop diuretic concomitantly with a QTc prolonging TKI may benefit from more close monitoring and correction of electrolyte abnormalities.

Strengths of our analysis include the real-world, community-based setting and the inclusion of patients receiving only those drugs with a risk for QTc prolongation. Additionally, individual risk factors and the use of concomitant QTc prolonging medications were assessed; however, these coadministrations occurred infrequently due to the proactive review and management by a pharmacist embedded within the oncology clinic. Our work is limited by the small number of patients in the analysis which reduces the power to make associations and the fact that not all previously identified risk factors were included. The small sample size was largely a result of a lack of follow-up ECG which have only more recently become the standard of care for patients receiving these medications. The fact that this was a retrospective and single center analysis is also a limitation; however, this does provide insight into the practice of monitoring and managing patients in the community setting. Additionally, as demonstrated by the number of patients without a follow-up ECG and therefore excluded from this analysis, there is a possibility of selection bias and an over estimation of the true incidence of QTc prolongation. Despite these limitations, when comparing the risk factors included, the groups were very similar apart from the more frequent utilization of loop diuretics in the group experiencing QTc prolongation.

## Conclusion

This real-world analysis of TKIs known to prolong the QTc interval in a community-based oncology clinic demonstrated a potentially higher incidence of QTc prolongation compared to prior studies. However, despite nearly a quarter of patients with observed QTc prolongation, no patients discontinued therapy primarily because of QTc prolongation. It has been recently recommended that patients receiving QTc prolonging TKIs be screened at baseline and either once steady state has been reached or after 14 days of therapy. Due to the potential increase in risk among patients receiving concomitant loop diuretics, it may be prudent to monitor patients more closely for QTc prolongation and electrolyte abnormalities. Further study is warranted to investigate the potential association between QTc prolongation and the use of loop diuretics, as well as the impact of other potential risk factors for QTc prolongation in the ambulatory oncology setting.

## Data availability statement

The raw data supporting the conclusions of this article will be made available by the authors, without undue reservation.

## Ethics statement

The studies involving human participants were reviewed and approved by IRB00011381 - Franciscan Health IRB #1. Written informed consent for participation was not required for this study in accordance with the national legislation and the institutional requirements.

## Author contributions

These authors share first authorship: DR, MR. This author served in the senior authorship role: VR. All authors developed and approved of the retrospective study protocol. MR completed data collection. MR and DR completed data analysis. VR provided input into data analysis. DR created the first draft of the manuscript with input from VR and MR. VR provided critical input into manuscript revision. All authors contributed to the article and approved the submitted version.
